# Supplementary material for: Genome-wide analysis provides genetic evidence that ACE2 influences COVID-19 risk and yields risk scores associated with severe disease
Source: Nat Genet. 2022 Mar 3;54(4):382–92. doi: 10.1038/s41588-021-01006-7 (PMC9005345; doi:10.1038/s41588-021-01006-7)
Supplement: Supplementary file 1 — Reporting Summary [file 41588_2021_1006_MOESM1_ESM.pdf]

Corresponding author(s): Manuel A. R. Ferreira

Last updated by author(s): September 22, 2021

Reporting Summary

Nature Research wishes to improve the reproducibility of the work that we publish. This form provides structure for consistency and transparency in reporting. For further information on Nature Research policies, see our Editorial Policies and the Editorial Policy Checklist.

Statistics

For all statistical analyses, confirm that the following items are present in the figure legend, table legend, main text, or Methods section.

n/a	Confirmed	
☐	☒	The exact sample size (*n*) for each experimental group/condition, given as a discrete number and unit of measurement
☐	☒	A statement on whether measurements were taken from distinct samples or whether the same sample was measured repeatedly
☐	☒	The statistical test(s) used AND whether they are one- or two-sided<br>*Only common tests should be described solely by name; describe more complex techniques in the Methods section.*
☐	☒	A description of all covariates tested
☐	☒	A description of any assumptions or corrections, such as tests of normality and adjustment for multiple comparisons
☐	☒	A full description of the statistical parameters including central tendency (e.g. means) or other basic estimates (e.g. regression coefficient) AND variation (e.g. standard deviation) or associated estimates of uncertainty (e.g. confidence intervals)
☐	☒	For null hypothesis testing, the test statistic (e.g. *F*, *t*, *r*) with confidence intervals, effect sizes, degrees of freedom and *P* value noted<br>*Give P values as exact values whenever suitable.*
☒	☐	For Bayesian analysis, information on the choice of priors and Markov chain Monte Carlo settings
☒	☐	For hierarchical and complex designs, identification of the appropriate level for tests and full reporting of outcomes
☒	☐	Estimates of effect sizes (e.g. Cohen's *d*, Pearson's *r*), indicating how they were calculated

Our web collection on statistics for biologists contains articles on many of the points above.

Software and code

Policy information about availability of computer code

Data collection

Data transfer from sequencing machine to DNAnexus
-Upload Agent v1.5.30 https://wiki.dnanexus.com/Downloads#Upload-Agent

Single-sample processing, all in DNAnexus
-Conversion of sequencing data in BCL format to FASTQ format and the assignments of paired-end sequence reads to samples based on 10-base barcodes; bcl2fastq v2.19.0 https://support.illumina.com/sequencing/sequencing_software/bcl2fastq-conversion-software.html
-Read alignment; bwa 0.7.17 http://bio-bwa.sourceforge.net
-Duplicate marking, stats gathering; picard v1.141 https://broadinstitute.github.io/picard/
-SAM/BAM/CRAM file generation and manipulation; samtools v1.7 http://www.htslib.org
-Variant calling; WeCall v1.1.2 https://github.com/Genomicsplc/wecall
-Sequence Quality Control; FastQC 0.11.8 http://www.bioinformatics/babraham.ac.uk/projects/fastqc/
-VCF file manipulation and index generation; bcftools v1.7 http://www.htslib.org, bgzip/tabix v1.7 http://www.htslib.org
-Multi-threaded file compression and decompression; pigz v2.3.4 https://zlib.net/pigz/
-haplotyping (Ancestry.com); Eagle v2.4.1 https://github.com/poruloh/Eagle
-imputation (Ancestry.com): Minimac4 v1.01 https://github.com/statgen/Minimac4

Generation of "freeze" data
-Joint genotyping to generate project-level VCF (pVCF) files; GLnexus v0.4.0 https://github.com/dnanexus-rnd/GLnexus
-Generation of variant representations in PLINK format; PLINK v1.90b6.21 https://www.cog-genomics.org/plink2/
-Ancestry predictions, IBD (Identity-by-descent) estimate, and pedigree reconstruction; PLINK v1.90b6.21 https://www.coggenomics.org/plink2/, PRIMUS https://primus.gs.washington.edu/primusweb/

Data analysis

- association testing: REGENIE v2.0.1 https://github.com/rgcgithub/regenie.

Data analysis	- meta-analysis: METAL (2020-05-05) https://github.com/statgen/METAL.<br>- various: python v3.8 https://www.python.org/downloads/; R v4.0.4 https://cran.r-project.org

For manuscripts utilizing custom algorithms or software that are central to the research but not yet described in published literature, software must be made available to editors and reviewers. We strongly encourage code deposition in a community repository (e.g. GitHub). See the Nature Research guidelines for submitting code & software for further information.

Data

Policy information about availability of data

All manuscripts must include a data availability statement. This statement should provide the following information, where applicable:

- Accession codes, unique identifiers, or web links for publicly available datasets
- A list of figures that have associated raw data
- A description of any restrictions on data availability

Full genotype-phenotype association results reported in this study are freely available for browsing and download using the Regeneron Genetics Center (RGC)'s COVID-19 Results Browser (https://rgc-covid19.regeneron.com). Data access and use is limited to research purposes in accordance with the Terms of Use (https://rgc-covid19.regeneron.com/terms-of-use); and agree that any public use of the Data must cite the associated publication, disclose that the RGC was the source of the data, and make the following statement: "The Regeneron Genetics Center bears no responsibility for the analyses or interpretations of the data presented here. Any opinions, insights, or conclusions presented herein are those of the authors and not of The Regeneron Genetics Center." as well as include the RGC name and logo on any public disclosure of the Data.. Users of the data may not attempt to identify any individuals who are subjects of the Data; combine the Data with other data in a manner that could lead to identification of an individual; copy or use the Data in any manner except as expressly permitted by this Agreement; directly commercialize the Data, including but not limited to, any sale, lease, license or transfer of the Data for monetary or other commercial gain; reverse engineer, disassemble, or decompile the Data; alter or remove any proprietary notices in the Data; or use or make available the Data for any purpose that is unlawful. For further questions on data access and restrictions, please contact rgc-covidrb@regeneron.com and we will respond in a timely manner.

Field-specific reporting

Please select the one below that is the best fit for your research. If you are not sure, read the appropriate sections before making your selection.

☒ Life sciences ☐ Behavioural & social sciences ☐ Ecological, evolutionary & environmental sciences

For a reference copy of the document with all sections, see nature.com/documents/nr-reporting-summary-flat.pdf

Life sciences study design

All studies must disclose on these points even when the disclosure is negative.

Sample size	Sample sizes were all those available in UK Biobank, Penn Medicine Biobank, Ancestry.com, or Geisinger Health Systems as described in the text. No power calculations were performed or required in advance.
Data exclusions	Prior to any analysis, we established the following data exclusions: We excluded individuals that were not predicted to belong to 5 continental ancestry groups (AFR, AMR, EAS, EUR, SAS) and furthermore did not analyze sets of individuals with fewer than 25 cases and 25 controls.
Replication	No replication was performed. Because we contributed our data to the Host Genetics Initiative of COVID-19, we could not use their summary statistics for replication (as our samples overlapped). Furthermore, the main novel finding, a protective variant in ACE2, was below the minimum allele frequency (5% or 1%) used by other studies and thus was not present in external summary statistics preventing replication.
Randomization	We performed a GWAS, which was an observational study, and as such no process of randomization was performed or applicable here because there was no allocation of samples into experimental groups.
Blinding	We performed a GWAS, which was an observational study, using coded de-identified data. As such, no process of blinding to group allocation was performed or applicable here.

Reporting for specific materials, systems and methods

We require information from authors about some types of materials, experimental systems and methods used in many studies. Here, indicate whether each material, system or method listed is relevant to your study. If you are not sure if a list item applies to your research, read the appropriate section before selecting a response.

Materials & experimental systems

n/a	Involved in the study
☒	☐ Antibodies
☒	☐ Eukaryotic cell lines
☒	☐ Palaeontology and archaeology
☒	☐ Animals and other organisms
☐	☒ Human research participants
☒	☐ Clinical data
☒	☐ Dual use research of concern

Methods

n/a	Involved in the study
☒	☐ ChIP-seq
☒	☐ Flow cytometry
☒	☐ MRI-based neuroimaging

Human research participants

Policy information about studies involving human research participants

Population characteristics	Population characteristics can be found in Supplementary Tables 1-3.
Recruitment	UK Biobank recruited approximately 500,000 individuals 40-69 years of age in 2006 to 2010 by mailers to people in the UK medical system. Informed consent was obtained for all participants. AncestryDNA customers over age 18, living in the United States, and who had consented to research, were invited to complete a survey assessing COVID-19 outcomes and other demographic information including SARS-CoV-2 swab and antibody test results, COVID-19 symptoms and severity, brief medical history, household and occupational exposure to SARS-CoV-2, and blood type. Geisinger Health System (GHS). The GHS MyCode Community Health Initiative is a health system-based cohort from central and eastern Pennsylvania (USA) with ongoing recruitment since 2006. Penn Medicine BioBank (PMBB) study. PMBB study participants are recruited through the University of Pennsylvania Health System, which enrolls participants during hospital or clinic visits. Any known biases from EHR-based datasets, survey datasets, or population-based datasets may be present and applicable here to this study and it's results.
Ethics oversight	Ethical approval for the UK Biobank was previously obtained from the North West Centre for Research Ethics Committee (11/NW/0382). The work described herein was approved by UK Biobank under application number 26041. GHS study: approval for DiscovEHR analyses was provided by the Geisinger Health System Institutional Review Board under project number 2006-0258. AncestryDNA study: all data for this research project was from subjects who provided prior informed consent to participate in AncestryDNA's Human Diversity Project, as reviewed and approved by our external institutional review board (Pro00034516), Advarra (formerly Quorum). All data was de-identified prior to use. PMBB study: appropriate consent was obtained from each participant regarding storage of biological specimens, genetic sequencing and genotyping, and access to all available EHR data. This study was approved by the Institutional Review Board of the University of Pennsylvania and complied with the principles set out in the Declaration of Helsinki.

Note that full information on the approval of the study protocol must also be provided in the manuscript.
